# Use of Reporter Genes in the Generation of Vaccinia Virus-Derived Vectors

**DOI:** 10.3390/v8050134

**Published:** 2016-05-21

**Authors:** Sally Al Ali, Sara Baldanta, Mercedes Fernández-Escobar, Susana Guerra

**Affiliations:** Department of Preventive Medicine, Public Health and Microbiology, Universidad Autónoma, E-28029 Madrid, Spain; sally.alali@yahoo.com (S.A.A.); sara.baldanta@uam.es (S.B.); mercedes.fernandeze@uam.es (M.F.-E.)

**Keywords:** vaccinia virus, vaccine, reporter-expressing virus

## Abstract

Vaccinia virus (VACV) is one of the most extensively-studied viruses of the *Poxviridae* family. It is easy to genetically modify, so it has become a key tool for many applications. In this context, reporter genes facilitate the study of the role of foreign genes introduced into the genome of VACV. In this review, we describe the type of reporter genes that have been used to generate reporter-expressing VACV and the applications of the recombinant viruses obtained. Reporter-expressing VACV are currently employed in basic and immunology research, in the development of vaccines and cancer treatment.

## 1. Introduction

Since the first description of recombinant DNA techniques, many advances have been achieved in the field of molecular biology and genetic modification. Currently, there is a wide variety of tools that allow the genetic modification of animals, plants, bacteria and viruses [1,2,3,4]. The genetic modification of viruses has become one of the best strategies for introducing nucleic acids into different cells, tissues or even in *in vivo* models, given the high transfection efficiency and ease of carrying it out, compared to chemical or physiological methods [5,6].

After the description of recombination events in cells infected with vaccinia virus (VACV) and through recombinant DNA technology [7,8], VACV has become a suitable model for the generation of recombinant virus vectors [9]. At first, the main purpose for introducing foreign genes into virus genomes was basic research about the biology of the viruses both *in vitro* and *in vivo*. However, with the latest technical advances and the higher understanding of the VACV viral cycle, virus genetic modification is getting a wider spectrum purpose. Thus, they can also be used for the development of vaccines or as oncolytic agents. This review aims to highlight the main aspects of the genetic modification of VACV and the generation and application of reporter-expressing virus in this model.

## 2. Biology of VACV

VACV is the prototype member of the *Poxviridae* family, so most research of poxvirus has been focused on its use [10]. VACV is a large DNA double-stranded virus, with a complex envelope. It was the live vaccine used to eradicate smallpox and nowadays is also used as a viral vector for recombinant vaccines and cancer therapy [9,11]. The VACV genome is one of the largest of all DNA viruses, with a size of 190 kbp and about 250 encoding genes [12]. The genome has a high genetic compaction, with a few intergenic and small non-coding regions. The coding regions are continuous, thereby not given to splicing [13,14].

**Figure 1:**
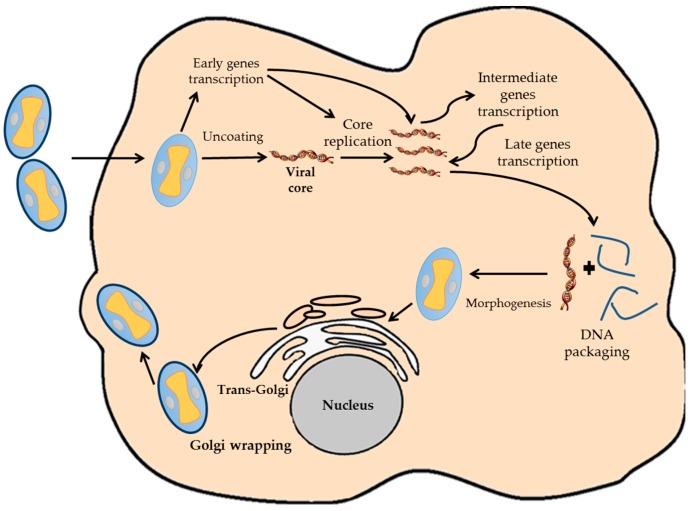
Diagram representative of the VACV infection cycle. The different steps of the VACV cycle are indicated.

VACV have a complete replicating cycle inside the cytoplasm of the host cell, even though it is a DNA virus (Figure 1) [10]. This fact determines the genetic characteristics of the virus, being completely independent of the replication and transcription machinery of the host cell. Once the virion infects the host cell, the viral core is uncoated, and nearly 100 early viral genes are transcribed [15,16]. Early genes produce the required enzymes for catalyzing the viral core breakdown, viral DNA replication and the modulation of the host antiviral response [17]. Viral DNA begins to replicate inside the infected host cell using viral enzymes at 3 h post-infection. As soon as the viral replication starts, transcription of downstream genes encoding for regulatory proteins that induce the expression of the late genes occurs. Late genes encode for proteins and enzymes required for the assembly of new viral particles. After DNA and all viral proteins are synthesized, the process known as morphogenesis begins, which results in the formation of the new virions [18,19]. These can be retained inside the cell until cellular lysis or released to the environment by other mechanism [10,18].

## 3. VACV as a Vector

Several features of the biology of VACV make it suitable for its use as a vector in biological experiments, vaccine design or cancer therapy. The complete cytoplasmic replication of VACV facilitates the expression of foreign genes inserted in the viral genome and its detection or isolation [20,21]. Usually, bacterial or non-mammalian viral vectors fail to make the expressed proteins to perform its full activity as antigens. However, VACV has the ability to transcribe its genes using its own transcription factors and enzymes. That means that if a foreign gene is inserted directly to a VACV promoter element, it will be transcribed with foreign proteins reaching high levels of expression in the infected cell. Moreover, this replication cycle is an appropriate model for molecular and genetic investigations of *cis* and *trans* factors that are mainly required for gene expression [12,22]. Furthermore, since VACV remains in the cytoplasm, the risk of insertional mutagenesis and oncogenesis, the main problems encountered in gene therapy using integrative viruses, disappears. In some cases, patients treated with retroviral vectors have developed cancer years after they have been treated [5,23].

VACV can replicate in different cell lines, primary cell cultures, and also grows in several animal species, such as mice, guinea pigs, rabbits, *etc.* [10]. This broad host range allows infection of cell lines with recombinant viruses for large-scale expression of heterologous proteins, which reduces its cost in comparison to other production systems [21,24]. Additionally, VACV enables high production titers, so it is an advantage in the manufacturing of a large amount of vaccines [6].

Although the VACV genome is large and compact, it can tolerate the deletion of certain viral sequences and the insertion of exogenous genetic material [25]. A VACV vector has a transgene capacity of approximately 25–30 kb, higher than other viral vectors, including adeno-associated virus (4.5 kb), adenovirus (8–10 kb) and retrovirus (7–8 kb) [4]. Thus, VACV is an excellent candidate vector in the design of polyvalent vaccines with antigens from several pathogens or different antigens from the same pathogen [9,26].

Finally, as far as its use as a vaccine vector is concerned, VACV is clearly immunogenic effective, strong evidence being the eradication of smallpox in 1980 [11]. VACV is also safe and easy to inoculate, since it can be administrated intradermally or with an air gun without medical training. In some organisms, it has been found that it can cause problems by preexisting immunity, but the probability of having post-vaccination complications, such as progressive VACV infection or encephalitis, is significantly low[27]. Nowadays, due to the better knowledge of the VACV biology and the immune response generated after vaccination, vaccines based on this virus are becoming safer [9]. In addition, it is important to remark that VACV vectors are very stable and can be lyophilized and kept frozen for several years, facilitating its transport and storage [23].

## 4. Design Considerations in the Generation of VACV Vectors

**Figure 2:**
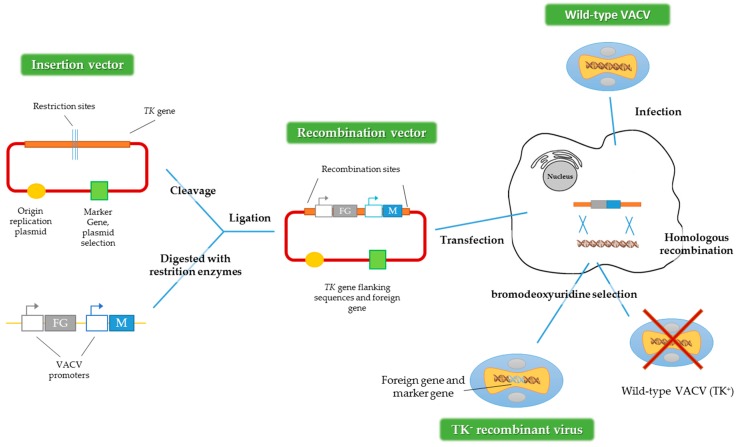
Construction of recombinant VACV vectors by homologous recombination. FG represents the foreign gene and M represents the marker gene and *TK*: *thymidine kinase* gene. Adapted from [28].

To get recombinant VACV expressing foreign genes, the main method used is homologous recombination (Figure 2) [28]. First, it is necessary to construct a plasmid that contains the gene or transgene of interest. After that, the cells have to be infected with the virus and subsequently transfected with the plasmid that contains the transgene. An alternative method could be used, employing two viruses, one defective for some genes and one wild-type acting as a helper [4,29]. For both methods, the recombinant viruses are produced by homologous recombination inside the infected cell.

**Figure 3:**
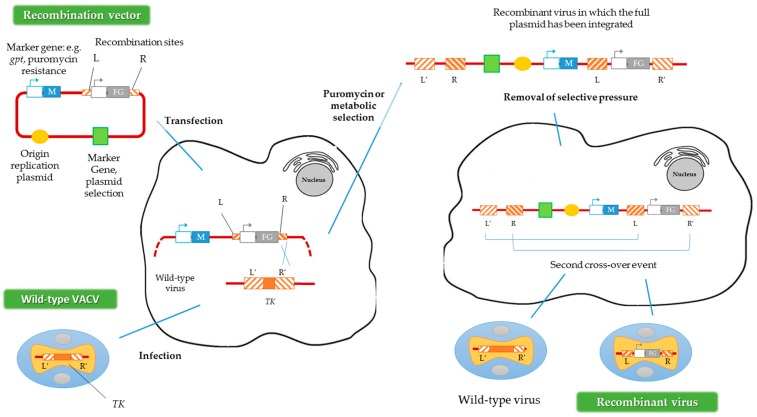
Construction of recombinant VACV vectors by the transient dominant selection (TDS) technique. FG represents the foreign gene and M represents the marker gene. R and L represent the right and left flanking regions of the *TK* gene in the plasmid, and R’ and L’ represent the same regions of the *TK* gene in the VACV genome. Adapted from [33].

Another way to generate recombinant viruses is the method described by Falkner and Moss [30], denominated transient dominant selection (TDS), which allows the introduction of site-directed mutations into the VACV genome. Generally, the recombinant viruses obtained by this method are rescued by metabolic selection, using the *guanine phosphoribosyltransferase* gene (*gpt*) from *Escherichia coli* as a marker. The presence of the protein encoded by *gpt* allows the recombinant viruses to grow in the presence of mycophenolic acid, xanthine and hypoxanthine [31]. Subsequently, after this first metabolic selection, a second recombination event must occur to eliminate the selection marker, maintaining the mutation introduced into the VACV genome (Figure 3) [32]. In contrast to the method described above, in the TDS technique the marker should not be flanked by homologous regions of the VACV genome [30]. Alternatively, puromycin resistance could be used as a selection marker in TDS, increasing the recombinant viruses’ generation efficiency [33].

Two important aspects to be considered when obtaining recombinant poxvirus are the VACV genome insertion sites and the reporter genes introduced.

### 4.1. VACV Genome Insertion Sites

**Figure 4:**
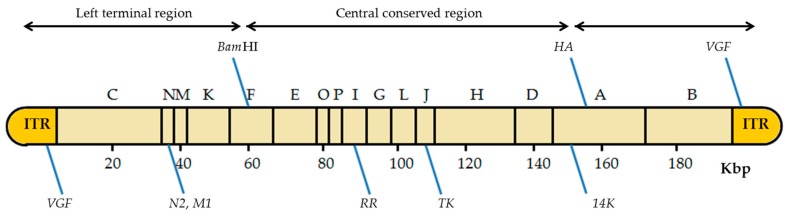
Scheme of the insertion sites in the VACV genome. The diagram of the VACV genome with the *Hin*dIII restriction sites is shown, including the location of the different insertion sites. *Bam*HI: *Bam*HI site of the *Hin*dIII F fragment; *HA*: hemagglutinin gene; *VGF*: *VACV growth factor* gene; *N2*: *N2* gene; *M1*: *M1* gene encodes the large subunit of ribonucleotide reductase (RR); *TK*: *thymidine kinase* gene; *A27L*: gene that encodes the 14 kDa fusion protein; ITRs: inverted terminal repeats. Adapted from [13].

The VACV genome has about seven known insertion sites where foreign genes can be inserted (Figure 4) [13]. The insertion site choice depends mainly on the future application of the recombinant viruses. It may also be important in the later selection of the recombinant viruses obtained. For instance, inserting the gene of interest in the *thymidine kinase* (*TK*) locus confers a detectable phenotype (TK-): the recombinant viruses are able to grow in the presence of 5-bromo-2’-deoxyuridine (BrdU), a synthetic analog of thymidine [28,34]. Another important site of insertion that allows a subsequent selection is the VACV *hemagglutinin* (*HA*) gene as the recombinant viruses can be easily recognized by their disability to bind erythrocytes in a hemagglutination test [35,36,37].

VACV has five more places of insertion: the *Bam*HI site of the *Hin*dIII-F DNA fragment [38]; the *VACV growth factor* gene (*VGF*), located in both inverted terminal repeats (ITRs) [39]; the *N2* and *M1* genes located on the left side of the VACV genome [40]; the M1 subunit of the *ribonucleotide reductase* (*RR*) gene in the *Hin*dIII-I DNA fragment [41]; and the *A27L* gene encoding the 14 kDa fusion protein, in the large *Hin*dIII-A DNA fragment [42]. It is noteworthy that some strains of VACV have only one copy of *VFG*, such as VACV Lister variants [33]. Recombinant production using these insertion sites, although successfully occurring, requires the use of a marker gene or other strategies for later selection of the recombinant viruses. Due to these limitations, the *TK* gene is the most common site of insertion in the VACV genome [5]. Some authors have used temperature-sensitive VACV strains, allowing the recombinant viruses to be selected in culture at 40 °C [43]. However, the most common way for an easy identification of recombinant viruses is the use of reporter genes as selectable markers, which will be discussed in Section 4.2 [44].

In spite of the promoter or the regions between the promoter and coding region, the insertion site also influences foreign gene expression and virus virulence [13,23,25]. Insertion into the *TK*, *VGF*, *RR* or *A27L* genes has an impact on viral replication *in vivo*, but not *in vitro* [25,45]. Moreover, the method described in Figure 2 requires the use of special cell lines or mutagenic selective agents, such as TK-/- cell lines and BrdU [30]. For this reason, different strategies and new insertion sites are being studied to ensure the correct expression of the transgenes *in vitro* and *in vivo* [25,33,46,47].

### 4.2. Reporter-Expressing Viruses

Reporter-expressing viruses are recombinant viruses expressing a reporter gene [48]. In some cases, the reporter gene is located downstream of a viral promoter, to study biological pathways or, fused with other viral or foreign genes. As reporter genes are expected to be easily detected, they are the best indicators for screening successfully recombinant viruses. The reporter gene should be chosen considering the non-endogenous activity in the cell type, tissue or organism used to culture the viruses [44]. Reporter genes have additional applications *in vitro* and *in vivo*, as the reporter gene acts as a substitute of the gene of interest. Moreover, reporter genes facilitate the use of tissue-specific and pathway-specific promoters, as well as regulatory promoter elements as biomarkers for a particular event route. Furthermore, it is important that the existence of the reporter gene should not affect the normal physiology and general characteristics of the transfected cells [48,49,50]. Table 1 presents an overview of the reporter genes commonly used in the generation of recombinant VACV.

#### 4.2.1. Chloramphenicol Acetyltransferase

*CAT* was the first reporter gene used in transcriptional assays in mammalian cells. CAT is an enzyme from *Escherichia coli* that detoxifies the antibiotic chloramphenicol, which inhibits protein synthesis in bacteria [58]. Particularly, CAT links acetyl-coenzyme A (acetyl-CoA) groups to chloramphenicol, preventing it from blocking the 50 S ribosomal subunit. This gene is not found in eukaryotes, so eukaryotic cells do not present any basal CAT activity [44]. The reaction catalyzed by CAT can be quantified using fluorogenic or radiolabeled substrates, such as ^3^H-labeled acetyl-CoA and ^14^C-labeled chloramphenicol. CAT can be detected either by thin-layer chromatography, autoradiography or enzyme-linked immunosorbent assay (ELISA) [51].

There is a strong link between *CAT* gene transcript levels and enzymatic activity, which is easy to quantify. Thus, *CAT* has become a suitable reporter gene for investigating transcriptional elements in a wide variety of experiments implicating animal and plant cells, as well as viruses [51]. There are some disadvantages of using the CAT system, such as the higher amount of cells required when compared to other assays, like the luciferase assay (detailed in Section 4.2.5). In addition, the CAT system is not suitable for use with weakly-expressed genes and *CAT* promoter activity quantification takes longer than other reporter systems [52]. Finally, this reporter gene has another important limitation due to the use of radioisotopes [44].

#### 4.2.2. β-Galactosidase

The first study using *lacZ* as a reporter gene was published in 1980, and since then, it has become one of the most commonly-used reporter genes in molecular biology [49]. Although β-galactosidase catalyzes the cleavage of the disaccharide lactose to form glucose and galactose, it recognizes several artificial substrates, which has promoted its use as a reporter gene [58]. Thus, β-galactosidase can hydrolyze substrates such as ortho-nitrophenyl beta-galactoside (ONPG), 5-bromo-4-chloro-3-indolyl beta-D-galactopyranoside (X-Gal) and 3,4-cyclohexenoesculetin beta-D-galactopyranoside (S-Gal), resulting in a yellow, blue or black product precipitate, respectively [53,59]. Furthermore, expression of the *lacZ* gene can be stimulated with isopropyl beta-D-thiogalactopyranoside (IPTG), which is a highly stable synthetic and non-hydrolyzable analog of lactose [49].

One of the applications of the *lacZ* reporter gene is the selection of transformed bacterial colonies. The recombinant (white) and non-recombinant (blue) bacteria are discriminated based on the interruption of the *lacZ* gene by the insert DNA or gene of interest using X-Gal as a substrate [53]. Other uses are the visualization of the β-galactosidase expression in transfected eukaryotic cells or the selection of the recombinant virus by viral plaque screening [60]. Finally, *lacZ* is used to detect β-galactosidase activity in immunological and histochemical experiments [44]. One of the main advantages of using this reporter gene system is its low cost, since it does not require specific devices to detect the colorimetric reaction or to identify its expression.

#### 4.2.3. β-Glucuronidase

Another *Escherichia coli*-derived hydrolyzing enzyme gene that lends a reporter assay is *GUS*. The β-glucuronidase protein catalyzes the breakdown of complex carbohydrates, such as glycosaminoglycans. This reporter gene system has been widely used in transgenic plants, and it has also been successfully used in mammalian cells for VACV recombinant virus selection [54]. For the β-glucuronidase (GUS) assay 4-methylumbelliferyl beta-D-glucuronide (MUG) or 5-bromo-4-chloro-3-indolyl beta-D-glucuronide (X-Gluc) can be used as substrates. They respectively lead to a fluorogenic or a blue product after cleavage [61,62]. Monitoring β-glucuronidase activity through a GUS assay allows the determination of the spatial and temporal expression of the gene of interest [63].

#### 4.2.4. Florescent Proteins

The most known fluorescent protein is green fluorescent protein (GFP), which was cloned from the species of jellyfish *Aequorea victoria*. Because of the great impact of fluorescent proteins in molecular biology applications, the Nobel Prize in Chemistry 2008 was awarded to Osamu Shimomura, Martin Chalfie and Roger Y. Tsien for the discovery and development of GFP [64,65]. *GFP* is the most used reporter gene; however, genetic engineering has developed a wide variety of color mutants, such as red fluorescent protein (RFP) or yellow fluorescent protein (YFP) among others [49]. 

Fluorescent proteins tolerate N- and C-terminal fusions to a wide-range of proteins, have been expressed in most known cell types and are used as a non-harmful fluorescent marker in living cells and organisms. The use of fluorescent proteins allows a variety of applications: cell lineage tracker , reporter for gene expression assays or measure of protein-protein interactions. Additionally, cell-fixation is not needed to examine its expression, and the probability of artifacts is quite small compared to immunocytochemical methods which require cell fixation [44]. One of the disadvantages of these proteins is their size. Therefore, in some cases, they can affect the *in vivo* function of fused proteins or genes of interest. Nevertheless, one limitation of using GFP is its low sensitivity [66], another is that its signal cannot be exogenously amplified [50].

#### 4.2.5. Luciferases

The first luciferase (LUC), from the firefly *Photinus pyralis*, was cloned in 1980 and *LUC* has been widely used as a reporter gene. Later, it was also described in bacteria and dinoflagellates [44]. Luciferases are enzymes that catalyze a chemical reaction resulting in the production of light. Firefly luciferase oxidize the D-luciferin, in the presence of oxygen and adenosine triphosphate (ATP) as the energy source. As in β-Galactosidase assays, an exogenous substrate is needed, and it may be a disadvantage [49]. In other systems, such as the luciferase identified in bacteria (*lux*CDABE operon), the enzyme catalyzes the oxidation of long-chain aldehydes and flavin mononucleotides (FMNH_2_) in the presence of oxygen to yield green-blue light [67]. Although in bacteria this operon encodes all components necessary for light emission, it is limited in mammalian cells. Therefore, the exogenous substrate has to be added to improve the reaction [56]. Besides the different substrates required, each luciferase system is categorized by having specific kinetics, with a particular detection and sensitivities that require adjusting the experimental design [58,67].

The use of luciferase is extremely widespread in biological systems studies and includes cell proliferation assays, protein folding/secretion analyses, *in vivo* imaging and control of *in vivo* viral spreading [57,67,68,69]. The main advantage of using this system is its high sensitivity when compared to other systems, such as CAT. Additionally, the LUC system is more direct, rapid and suitable when it comes to weakly-expressed genes, and it can be used to quantify gene activity. One disadvantage of the LUC system is the requirement of ultrasensitive charge-coupled device (CCD) cameras to detect gene expression [56].

## 5. Applications of Reporter-Expressing Viruses

### 5.1. In Vitro Applications

Reporter-gene assays have helped the pox virologists in basic research, for example for the study of the location, structure and function of many VACV proteins during the infection cycle and their interaction with proteins of the host cell [44,70]. As shown in Dvoracek and Shors [63], the *GUS* reporter gene was used for deleting the D9R viral protein and selecting the recombinant viruses, with the aim to understand the role of this protein in the viral life cycle. In addition, the *lacZ* gene has typically been used mainly for the selection of recombinants [71]. Moreover, several studies have reported the different transgenes’ insertion points and VACV promoters in which the recombinant virus production was enhanced. These studies are essential for improvement of the development of vaccines based on recombinant VACV [62,72].

On the other hand, fluorescent markers such as the GFP, YFP or luciferase are also useful for labelling VACV replicative strains. These viruses have allowed the study of processes like the input and output morphogenesis in virus-infected cells [68,69,73,74,75,76]. In these studies, fluorescence of certain viral proteins allows us to study their interaction with other viral or cellular proteins [77]. Furthermore, VACV is a clear example of how viruses have developed strategies to evade the immune response [78]. In this field, the generation of recombinant VACV with reporter genes is also useful to discern the molecular mechanism by which VACV proteins manipulate the immune system of the host. Thus, in Unterholzner *et al.* [79], the generation of a GFP-labeled recombinant VACV revealed that the C6 viral protein acted as an immunomodulatory agent, blocking the expression of type I interferon. 

Another major application of reporter-expressing VACVs is the design of high-throughput assays. The generation of *lacZ* or *GFP* expressing recombinant virus can be used to optimize antibody neutralization assays [71,80].

Lastly, VACV and reporter genes have been used to study proteins from other viruses, particularly RNA viruses, such as influenza or severe acute respiratory syndrome-associated coronavirus (SARS-CoV) [81]. To genetically modify these viruses, RNA must be reverse transcribed to cDNA, since this is particularly unstable in plasmids, making VACV a good tool for functional studies of proteins from such viruses [82].

### 5.2. In Vivo Applications

There are several *in vivo* applications for recombinant reporter-expressing viruses. For example, in virulence studies, the use of labeled viruses allows us to follow the viral pathogenicity and detect in which organs the viral replication and dissemination occur [70,76]. For example, Zaitseva *et al.* [69] used the recombinant VACV Western Reverse strain (WR)-LUC to analyze the viral spread *in vivo* for several days reducing the number of mice used. Moreover, VACV is an effective enhancer for both humoral and cell-mediated immunity; it is used as a vector to study the immune system and the expression of proteins’ antigenicity of other pathogens. Furthermore, VACV is used to explore the immunopathological mechanisms, to know which epitopes or antigens presented by a pathogen have the ability to induce the host-immune response, and to demonstrate the specific role of a particular antigen during the pathogenic process [13,83,84].

Despite the examples mentioned above, the most common uses of recombinant VACV *in vivo* are the production of prophylactic vaccines and treatments against cancer [4,85]. Table 2 shows some of the vaccines based on VACV, with the reporter gene and the insertion site employed indicated in each case. In these vaccines, VACV acts as a vector capable of delivering antigens from other organisms [23]. While in many recombinant vaccines a viral antigen has been inserted, some of them have also been developed against bacteria [86] or protists [34,87,88]. These vaccines simulate infection by the pathogen from which the antigens are and elicit the immune response, by producing antigens for different pathogens. In several vaccines, mainly against human immunodeficiency virus (HIV) or influenza, genes of immunomodulatory cytokines are added for coexpression with the antigen, improving the immunogenicity of the vaccines [23,89,90]. As summarized in Table 2, most of the transgene insertion sites are within the *TK* or the *HA* genes, making the selection of recombinants easier, as explained above. However, in several vaccines, besides using this strategy, a reporter gene is used as well. The use of reporter genes facilitates the preliminary tests of the vaccine on animal models. Moreover, especially in vaccines used in animals, the reporter gene makes it possible to distinguish between vaccinated and infected animals [48]. For example, VACV has been used for nearly twenty years to eradicate rabies from wildlife as an oral-based vaccine. In this case, the recombinant VACV expresses the rabies virus glycoprotein and has been used to vaccinate raccoons, red foxes, skunks and coyotes in the United States and Europe. This battle has successfully purged rabies in some parts of Europe and the United States [91].

Another application for VACV vectors is in cancer treatment, known as oncolytic virotherapy [26]. This is the use of replication-competent viruses to selectively attack and destroy cancer cells, without harming healthy host cells [109]. Examples of recombinant VACV used are summarized in Table 3. A promising study is the use of oncolytic VACV as a vector for the *human sodium iodide symporter* (*hNIS*) gene in prostate cancer therapy, which has been demonstrated to restrict tumor growth and to increase survival in mice [110]. VACV is also a promising therapeutic agent for pancreatic cancer [85], cholangiocarcinoma [111] and colorectal cancer [112]. It is worth mentioning that many of the viral vectors developed to treat tumors have several common characteristics. Generally, VACV oncolytic vectors have a deletion in the *TK* gene, essential for the pyrimidine synthesis pathway, which forces the virus to replicate in cells displaying a high amount of nucleotide pools, enhancing the viral tropism to cancer cells. Others have a deletion in the *VGF* gene, preventing non-infected cells from proliferation [109]. Furthermore, as in the development of vaccines, viral vectors are “armed” with genes that enhance the antitumor activity, the virus tropism or the immunoreactivity, to promote better tumor destruction, such as *granulocyte-macrophage colony-stimulating factor* (*GM-CSF*) or *erythropoietin* genes (enhanced virus; Table 3). Another particular feature is that many of these recombinants carry reporter genes, and thus viral replication can be monitored by non-invasive imaging methods [68,69,76,113].

## 6. Limitations of VACV Vectors

The main limitation of using VACV as a vector is the short-term gene expression, since it is a lytic virus killing the infected cells. Thus, gene expression will not last for more than 12–24 h post-infection [13,109]. Additionally, although for some applications it is an advantage, since VACV replicates completely in the infected cell cytoplasm, it is hard to use VACV to engineer nuclear gene replacement [23]. The other main disadvantage is the limited immunogenicity in individuals vaccinated against smallpox. This pre-existing immunity reduces the effectiveness of vaccines based on VACV, although some trials have overcome this problem by mucosal vaccination with vaccinia vectors [5]. The VACV safety profile should be considered because it has progressive complications especially with immunocompromised individuals [11]. These limitations primarily affect *in vivo* applications of VACV recombinants in vaccine development, so several attenuated strains of VACV are being generated [9].

## 7. New Perspectives

As for other viruses, the development of vaccines or oncolytic therapies based on VACV requires the understanding of its pathogenesis and biology. Despite improvements in the vectors’ design, such as the use of different promoters or insertion sites, homologous recombination has been almost exclusively the way to obtain VACV recombinants [45]. Homologous recombination requires the use of markers or reporter genes for selecting recombinants, which offers many disadvantages. Apart from the physical space needed for the marker gene, which is limited in therapeutic virus, the use of certain markers can introduce mutations or generate artifacts that are only found after an analysis of the generated virus. Sometimes, these problems cannot be detected *in vitro*, but are very important to overcome when these vectors are used *in vivo* on animal models [46,48].

In recent years, some strategies have been developed to avoid these risks using markers, or at least to remove them from the final recombinant VACV. Rice and colleagues [45] described a double selection method to improve the selection of recombinant VACV, so that a reporter or marker gene is not necessary. A helper virus is used to rescue a recombinant VACV and is subsequently grown in non-permissive cells to the helper virus; allowing the selection of a large percentage of recombinant virions. However, the method that has certainly had an enormous importance in the modification of genomes is the clustered regularly interspaced short palindromic repeats (CRISPR)/CRISPR-associated protein 9 (Cas9) system. Briefly, the CRISPR/Cas9 system consists of an endonuclease (Cas9) employing a guide RNA to generate a break in a target place of the genome, later to be repaired, either randomly or precisely using a specifically designed “restful” template [119]. The effectiveness of this system has been proven in different organisms, including viruses, such as herpes simplex virus (HSV) [120], hepatitis B virus (HBV) [121] and HIV [76]. Currently, this technique is starting to be used also in VACV [47]. For example, this system has achieved the deletion of VACV virulence genes, such as *A46L* and *N1L*. A46L and N1L are VACV intracellular proteins that inhibit nuclear factor-kappa B (NF-kB) activation, so it is undesirable that they were present in VACV vectors with therapeutic purposes [78]. Furthermore, given the efficiency of the method, “reparative” vectors with excisable marker genes have been designed. Therefore, recombinant viruses are effectively isolated, but eventually, the marker gene is eliminated [46]. Given the simplicity of recombinant VACV by the CRISPR/Cas9 system generation, an exponential increase of applications with better markers for basic research or without selectable markers for clinical application is expected [119,120].

## 8. Concluding Remarks

In conclusion, the development of recombinant viruses is a promising therapeutic advance in the biomedical field. In this sense, the use of reporter-expressing VACVs has become a fundamental tool for a number of applications, in basic research, vaccine design and cancer therapy. As many of these trials are still experimental, more information is required regarding the side effects of the viral treatment. Continuing efforts are necessary to develop new reporter-expressing VACVs that are safer and more effective for future therapies.

## Figures and Tables

**Table 1:** Reporter genes commonly used in the generation of recombinant vaccinia virus (VACV).

Reporter Gene	Origin	Product	Detection	Reference
*CAT*	*Escherichia coli*	Chloramphenicol acetyltransferase	Thin-layer chromatography autoradiography, ELISA	[51,52]
*LacZ*	*Escherichia coli*	β-galactosidase	Colorimetry	[53]
*GUS*	*Escherichia coli*	β-glucuronidase	Colorimetry or fluorescence	[54]
*GFP*	*Aequorea victoria* (jellyfish)	Green fluorescent protein	Fluorescence	[50,55]
*LUC* or *lux*CDABE	*Photinus pyralis* (firefly) and bacteria	Luciferase	Luminescence	[56,57]

ELISA: enzyme-linked immunosorbent assay.

**Table 2:** VACV-derived vaccines.

Pathogenic Agent		Antigen	Features		Reference
			Site of Insertion	Reporter Gene	
Viral	HIV	Env	*TK* or *HA* gene	*LacZ*, *LUC*	[90,92]
		Env (TAB 13)	*HA* gene	*LacZ*	[35]
		RT	Not mentioned	*LacZ*	[93]
	Hepatitis B virus	HBsAg	*TK* gene or *Bam*HI site	Not mentioned	[38,94,95]
		PreS2-S	*TK* gene	Not mentioned	[96]
		LS	*TK* gene	Not mentioned	[97]
		MS	*TK* gene	Not mentioned	[98]
	Herpes simplex virus 1	gD	*TK* gene or *Bam*HI site	Not mentioned	[38,99,100]
		gB	Not mentioned	Not mentioned	[101,102]
		gG	Not mentioned	Not mentioned	[103]
	Influenza	HA	*TK* gene	Not mentioned	[104]
		M1, NS1, NP, PB1, PA	*TK* gene	Not mentioned	[105]
Protist	*Plasmodium yoelii*	Circumsporozoite	*TK* gene	*LacZ*	[34]
	*Plasmodium knowlesi*	Sporozoite antigen	*TK* gene	Not mentioned	[88]
	*Plasmodium falciparum*	S antigen	*TK* gene	Not mentioned	[87]
	*Leishmania infantum*	LACK	*TK* and *HA* gene	*LacZ* and *GUS*	[106]
Animal	*Echinococcus granulosus*	E95 antigen	*TK* gene	*LacZ*	[107]
Bacterial	*Brucella abortus*	18-kDa antigen	*TK* gene	*LacZ*	[86]
	*Streptococcus pyogenes*	M protein	*TK* gene	Not mentioned	[108]

**Table 3:** Oncolytic vaccinia virus (VACV) developed for cancer treatment.

Virus	Target Cancer	Features		Reference
		Inactive Genes	Additional Genes	
**Initial virus**				
JX-594	Melanoma, hepatocellular carcinoma, colorectal cancer	*TK* inactive,	*LacZ* and *GM-CSF*	[112,114]
GLV-1h68	Colorectal cancer, prostate cancer, salivary gland carcinoma	*TK*, *HA* and *F14*.*5L* inactive	*GFP*, *LacZ* and *GUS*	[111]
vvDD	Sarcomas, neuroblastoma	*TK* and *VGF* inactive	*CD*	[115,116]
**Enhanced virus**				
GLV-1h153	Pancreatic cancer	GLV-1h68 expressing *hNIS*		[110]
GLV-1h210	Lung cancer	GLV-1h68 expressing *hEPO*		[117]
vvDD-SR-RFP	Sarcomas, neuroblastoma	*TK* and *VGF* inactive	*CD*, *RFP*, *SR*	[118]

## Abbreviations

acetyl-CoA: acetyl-coenzyme A
ATP: adenosine triphosphate
CAT: chloramphenicol acetyltransferase
CCD: charge-coupled device
CD: cytosine deaminase
CRISPR/Cas9: clustered regularly interspaced short palindromic repeats/CRISPR-associated protein9
Env: envelope
FMNH_2_: flavin mononucleotide
gB: glycoprotein B
gD: glycoprotein D
GFP: green fluorescent protein
gG: glycoprotein G
GM-CSF: granulocyte-macrophage colony-stimulating factor
gpt: guanine phosphoribosyltransferase gene
GUS: β-glucuronidase
HA: hemagglutinin
HBsAg: hepatitis B virus surface antigen
HBV: hepatitis B virus
hEPO: human erythropoietin
HIV: human immunodeficiency virus
hNIS: sodium iodide symporter
HSV: herpes simplex virus
IPTG: isopropyl beta-D-thiogalactopyranoside
ITRs: inverted terminal repeats
LACK: Leishmania homolog of activated C kinase
lacZ: β-galactosidase gene
LS: large surface protein
LUC: luciferase
M1: matrix 1
MS: middle surface protein
MUG: 4-methylumbelliferyl beta-D-glucuronide
NF-kB: nuclear factor-kappa B
NP: nucleoprotein
NS1: non-structural protein 1
PA: polymerase acidic
PB1: polymerase basic 1
RT: retrotranscriptase
ONPG: ortho-nitrophenyl beta-galactoside
RFP: red fluorescent protein
RR: ribonucleotide reductase
SARS-CoV: severe acute respiratory syndrome-associated coronavirus
S-Gal: 3,4-cyclohexenoesculetin beta-D-galactopyranoside
SR: somatostatin receptor
TDS: transient dominant selection
TK: thymidine kinase
TK-: TK-defective phenotype
VACV: vaccinia virus
VGF: vaccinia growth factor
WR: Western Reserve strain
X-Gal: 5-bromo-4-chloro-3-indolyl beta-D-galactopyranoside
X-Gluc: 5-bromo-4-chloro-3-indolyl beta-D-glucuronide
YFP: yellow fluorescent protein.
